# Doxorubicin‐loaded nanoparticle coated with endothelial cells‐derived exosomes for immunogenic chemotherapy of glioblastoma

**DOI:** 10.1002/btm2.10203

**Published:** 2020-12-03

**Authors:** Chao Zhang, Jian Song, Lei Lou, Xuejiao Qi, Lei Zhao, Bo Fan, Guozhu Sun, Zhongqiang Lv, Zhenzeng Fan, Baohua Jiao, Jiankai Yang

**Affiliations:** ^1^ Department of Neurosurgery, the Second Hospital of Hebei Medical University Shijiazhuang Hebei China

**Keywords:** blood brain barrier, doxorubicin, exosome‐coated doxorubicin‐loaded nanoparticle, glioblastoma, immunogenic cell death

## Abstract

Treatments of glioblastoma (GBM) have not been very effective, largely due to the inefficiency of drugs in penetrating the blood brain barrier (BBB). In this study, we investigated the potential of exosome‐coated doxorubicin (DOX)‐loaded nanoparticles (ENP_DOX_) in BBB penetration, inducing immunogenic cell death (ICD) and promoting survival of GBM‐bearing mice. DOX‐loaded nanoparticles (NP_DOX_) were coated with exosomes prepared from mouse brain endothelial bEnd.3 cells. ENP_DOX_ cellular uptake was examined. Penetration of ENP_DOX_ through the BBB was tested in an in vitro transwell system and a GBM mouse model. The effects of ENP_DOX_ in inducing apoptosis and ICD were assessed. Finally, the efficacy of ENP_DOX_ in the treatment of GBM‐bearing mice was assessed. ENP_DOX_ was taken up by bEnd.3 cells and could penetrate the BBB both in vitro and in vivo. In vitro, END_DOX_ induced apoptosis and ICD of glioma GL261 cells. Systemic administration of ENP_DOX_ resulted in maturation of dendritic cells, activation of cytotoxic cells, altered production of cytokines, suppressed proliferation and increased apoptosis of GBM cells in vivo and prolonged survival of GBM‐bearing mice. Our findings indicate that ENP_DOX_ may be a potent therapeutic strategy for GBM which warrants further investigation in clinical application.

## 1 INTRODUCTION

Glioblastoma (GBM) is a highly aggressive brain tumor with an extremely poor prognosis and a small rate (4%–5%) or 5‐year survival.^1^ Current treatments of GBM include surgeries, radiotherapy, and/or chemotherapy. These treatments not only cause severe side effects, but also only slightly improve the overall median survival (only 15 months) and 5‐year survival rate.^2^ Although many therapeutic strategies targeting have been developed, their application in the clinic for treatment GBM has been largely impeded due to the lack of safe and efficient drug delivery system that delivers drugs to tumor location.^3^ Recent research findings suggest that, in various cancer types including GBM, human immune response has significant potential in promoting immune mediated tumor eradication and improving long term survival.^4^

Recent studies have shown that anthracyclines, such as doxorubicin (DOX), not only induce apoptosis of tumor cells, but also immunogenic cell death (ICD).^5^ ICD is a special type of cell death that elicits immune responses, leading to maturation of dendritic cells and activation specific T cells, an outcome is that largely preferred in anticancer therapy.^6^ DOX treatment leads to translocation of calreticulin (CRT) from the endoplasmic reticulum to the plasma membrane surface, ATP secretion, and release of the nonhistone chromation protein high‐mobility group box 1 (HMGB1). These events are characteristics of ICD, the highly sought for goal in cancer therapy. DOX has been shown to cause cytotoxicity in various tumor cells and is currently used as treatment for different cancers. When delivered locally, DOX is able to improve the survival of rats bearing malignant intracranial glioma.^7^ Additionally, interstitial administration of DOX in patients with recurring malignant glioma who underwent repeated surgeries resulted in significant improvement with one patient showing no tumor recurrence following 2 months of treatment.^8^ However, it is not clear whether this route of drug delivery is safe and DOX was administered by insertion of a catheter into the location of removed tumor following surgery. Therefore, we aimed to explore a systemic drug delivery method that allows penetration of DOX through the blood brain barrier (BBB) with minimal invasiveness and that targets specifically to tumor cells.

Recent discovery of exosome‐mediated drug delivery has attracted great attention in the fields of neurological disorders, whose treatments are largely impeded by the inefficiency of drugs in penetrating the BBB, a natural barrier that separates the central nervous system from the peripheral circulation. Exosomes are membrane‐wrapped extracellular microvesicles with diameters of 40–200 nm that are secreted by most cell types. Exosomes contain multiple nucleic acids and proteins and play important roles in cellular communication. The membranes of exosomes have various membrane proteins with specific functions and may be involved in targeting exosomes to brain vascular endothelial cells and in penetration of the BBB. Exosomes have been shown to have great therapeutic potential in the treatment of various diseases, such as cancer, chronic obstructive pulmonary disease, and Alzheimer's disease.^9^, ^10^, ^11^ Recently, exosomes have been extensively studied as a vehicle for drug delivery. Exosomes are able to cross the BBB and have been use to carry specific siRNAs and mediate gene knock down in the brain.^12^ Exosome‐coated drug loading nanoparticles have been developed and its potential in treating breast cancer has been explored.^13^ Importantly, this drug‐loading method has been shown to successfully deliver DOX in breast cancer cells leading potent apoptosis of these cells and suppression of tumor growth.^13^ Although the effectiveness of DOX for treating GBM is largely impacted by its inefficiency in BBB penetration, it has been shown in vitro that DOX can effectively induce death of GBM cells and DOX‐coated nanoparticles can penetrate a model of BBB composed by a monolayer of Madin–Darby canine kidney transfected with multidrug resistant protein 1.^14^ Therefore, in this study, we took advantage of the exosome‐mediated drug delivery system and explored the potential of exosome‐coated DOX‐loaded nanoparticles in penetrating the BBB, in inducing apoptosis and ICD of tumor cells and in the survival of GBM‐bearing mice.

## 2 RESULTS

### 2.1 Design of exosome‐coated DOX‐loading nanoparticles for GBM treatment

**FIGURE 1:**
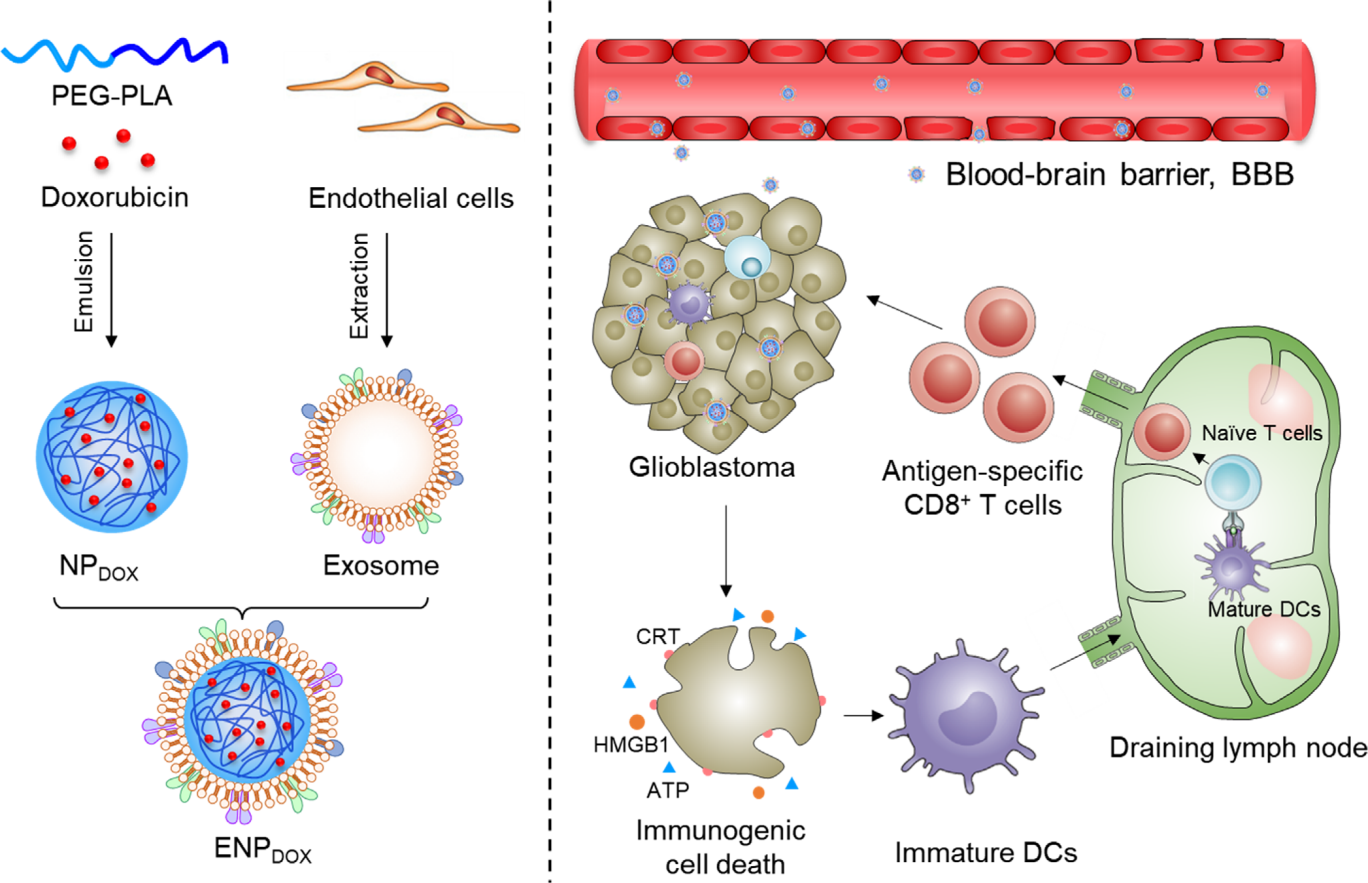
Schematic illustration of endothelial cells‐derived exosomes coated nanomedicine for glioblastoma treatment. Doxorubicin (DOX), which can induce immunogenic cell death (ICD), was encapsulated into polymeric nanoparticles via an emulsion method. To enhance the capability of blood–brain barrier (BBB) penetration, DOX‐loaded nanoparticles (NP_DOX_) were packaged into endothelial cell‐derived exosomes (ENP_DOX_). After intravenous administration, ENP_DOX_ can better penetrate BBB and accumulate in glioblastoma tissues. The enriched ENP_DOX_ induce apoptosis and immunogenic cell death of glioblastoma cells (including the exposure of calreticulin [CRT], the release of ATP, and high‐mobility group box 1 [HMGB1]), resulting in the maturation of dendritic cells and infiltration of cytotoxic T lymphocytes

To enhance the delivery of DOX to tumor area across the BBB in patients with GBM and induce antitumor immune response, we developed a drug delivery system by coating DOX‐loaded nanoparticles with exosomes isolated from bEnd.3 cells (a murine brain endothelial cell line) (Figure 1) according to a previous method.^13^ Briefly, PEG–PLA nanoparticles loading DOX (NP_DOX_) were prepared through an emulsion method.^15^ Exosomes were harvested from endothelial cell cultured and their contents were emptied. The NP_DOX_ were then packaged into the empty exosomes to form the exosome‐coated DOX‐loaded nanoparticles (ENP_DOX_). Following intravenous administration, ENP_DOX_ is expected to penetrate the BBB, accumulate at the GBM site and induce apoptosis and ICD, resulting in maturation of dendritic cells and infiltration of cytotoxic T lymphocytes (right panel, Figure 1).

### 2.2 ENP_DOX_ was taken up by endothelial cells in vitro

**FIGURE 2:**
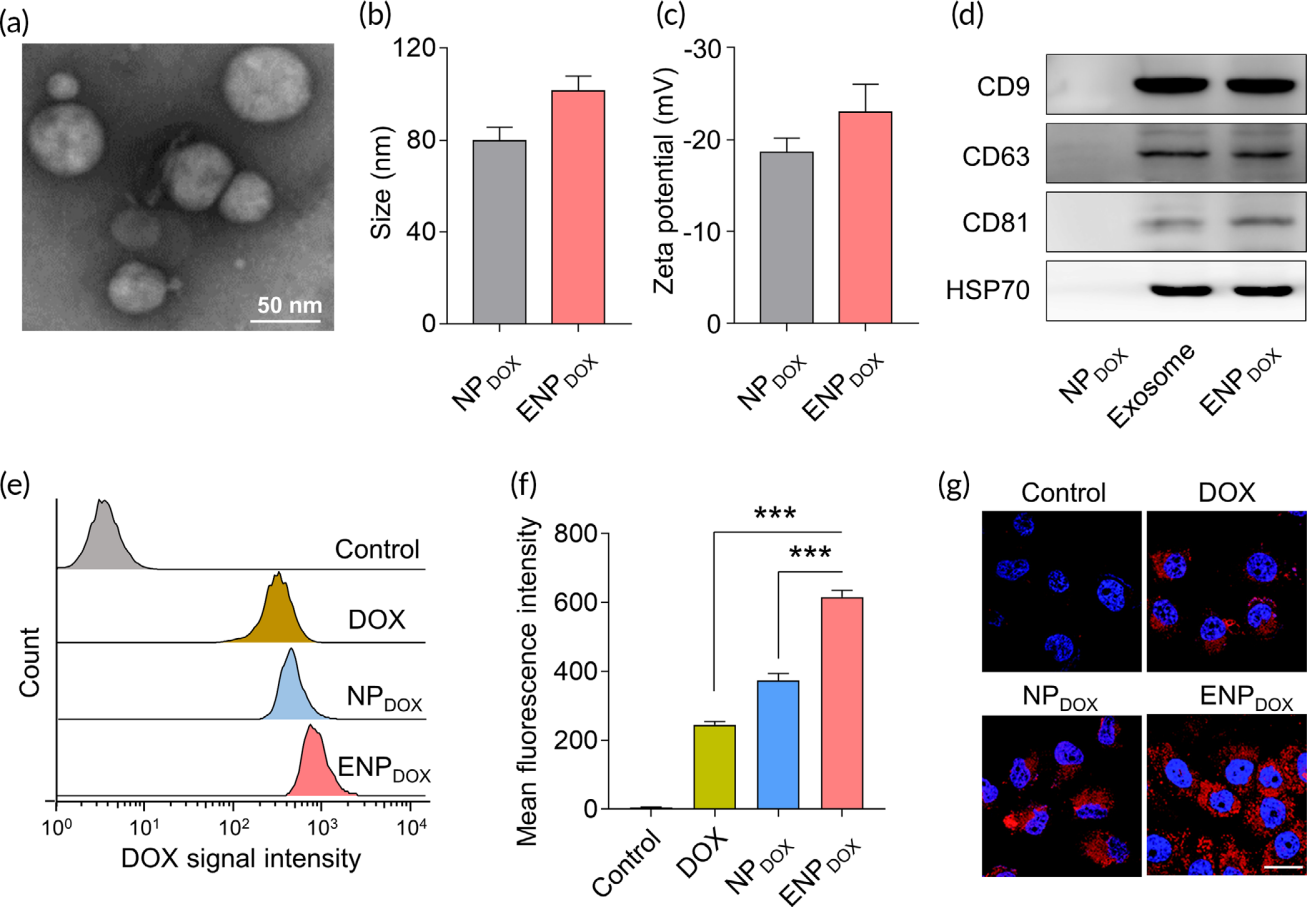
In vitro cellular uptake of endothelial cells. (a) Representative transmission electron microscopy (TEM) images of exosome derived from bEnd.3 cells (murine brain endothelial cell line). Scale bar: 50 nm. (b) The size distribution of doxorubicin (DOX)‐loaded nanoparticles (NP_DOX_) and exosome‐coated DOX‐loaded nanoparticles (ENP_DOX_). (c) Zeta potential of NP_DOX_ and ENP_DOX_. (d) Protein expressions on exosome, NP_DOX_, and ENP_DOX_ were examined via western blot. (e) Flow cytometry analysis of bEnd.3 cells incubated with DOX, NP_DOX_, and ENP_DOX_ with the same DOX concentration of 0.5 μg/mL for 4 h. (f) Quantification of the mean fluorescence intensities of the bEnd.3 cells. Data represent means ± SD. ***p < 0.001. (g) CLSM images of intracellular delivery of DOX, NP_DOX_, and ENP_DOX_ cells at 1 h. The nuclei were stained with DAPI. Scale bar: 20 μm

**FIGURE 3:**
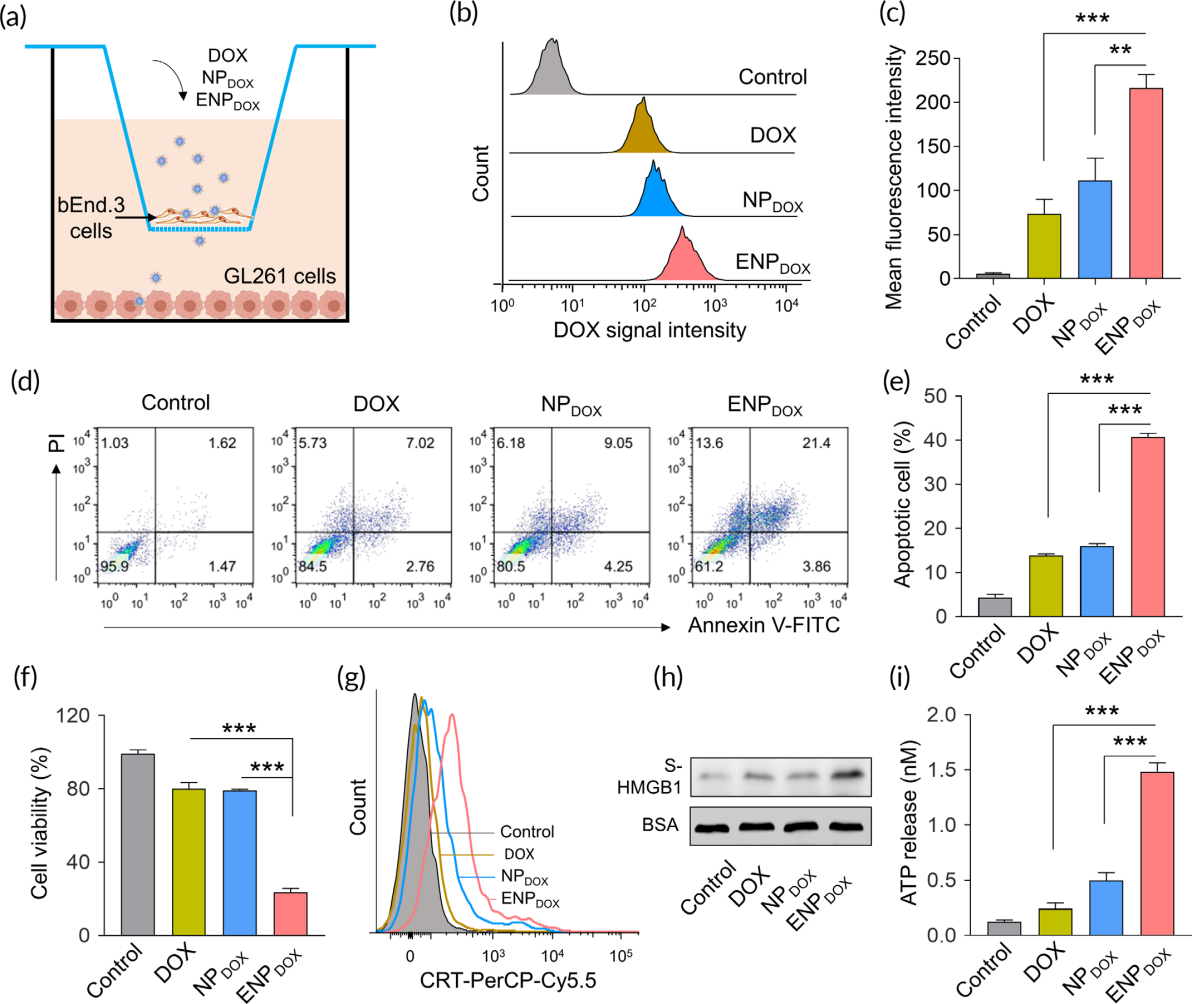
Exosome‐coated doxorubicin (DOX)‐loaded nanoparticles (ENP_DOX_) induces apoptosis and immunogenic cell death of murine glioma cells. (a) The transwell system was applied to measure the transport of DOX, ENP_DOX_, and ENP_DOX_ across the blood–brain barrier (BBB) in vitro. Murine brain endothelial cell line bEnd.3 and glioma cell line GL261 were cultured in the upper and lower compartment, respectively. The free DOX and nanoparticles were added to the upper compartment (0.5 mg/mL) for 12 h, and the cellular uptake of GL261 cells was examined. For the detection of apoptosis and ICD, GL261 cells were cultured for additional 24 h before examination. (b) Flow cytometry analysis of GL261 cells following transwell culture and treatment of DOX, DOX‐loaded nanoparticles (NP_DOX_), and ENP_DOX_ in the upper compartment. (c) Quantification of the mean fluorescence intensities of GL261 cells. Data represent means ± SD. **p < 0.01, ***p < 0.001. (d) GL261 cells were harvested for the detection of apoptosis by double staining with Annexin‐V and propidium iodide (PI) using flow cytometry. (e) The percentages of apoptotic GL261 cells. Data represent means ± SD. ***p < 0.001. (f) Cell viabilities detected by MTT assay. Data represent means ± SD. ***p < 0.001. (g) The surface exposure of calreticulin (CRT) was determined by flow cytometry among viable (propidium iodide negative) cells after treatment. DOX‐treated cells were stained with propidium iodide and FITC labeled anti‐CRT antibodies according to the manufacturer's instructions. (h) Released high‐mobility group box 1 (HMGB1) in the supernatant (S‐HMGB1) of GL261 cells treated with DOX, NP_DOX,_ and ENP_DOX_ was measured by western blot, and BSA was used as the loading control. (i) The amount of released ATP was determined by a chemiluminescent ATP Determination Kit. Data represent means ± SD. ***p < 0.001

We first analyzed the characteristics of ENP_DOX_. The morphology of the nanoparticles was examined by TEM (Figure 2(a)) and their size was measured (Figure 2(b)). ENP_DOX_ had slightly none significant increase in the average diameter compared to that of NP_DOX_. NP_DOX_ and ENP_DOX_ had similar zeta potential (Figure 2(c)). Examination of proteins on the surface of exosomes by Western blot analysis showed that packaging DOX‐loaded nanoparticles did not impact the expression of proteins on exosomes, including CD9, CD63, CD81, and HSP70 (Figure 3(d)).

We then determined the uptake of nanoparticles by mouse endothelial bEnd.3 cells. DOX fluorescent intensity was measured by flow cytometry analysis (Figure 2(e)). We found that cells treated with ENP_DOX_ showed significant increase in DOX fluorescent intensity (Figure 2(f)). We also examined DOX uptake by confocal imaging and found that DOX was more efficiently taken up by bEnd.3 cells (Figure 2(g)) compared to DOX or NP_DOX_ treatment.

### 2.3 ENP_DOX_ induces apoptosis and ICD of murine glioma cells in vitro

To assess the capability of ENP_DOX_ in penetrating the BBB, we first utilized an in vitro model of the BBB with a transwell system.^16^ In this culture system, bEnd.3 cells were cultured in the upper compartment and were treated with NP_DOX_ and ENP_DOX_, respectively, and GL261 cells were cultured in the lower compartment (Figure 3(a)). Examination of DOX signal intensity by flow cytometry (Figure 3(b)) showed that ENP_DOX_ treatment significantly enhanced DOX signal in GB261 cells compared to that of DOX or NP_DOX_ treatment (Figure 3(c)), indicating exosome coating promotes penetration of nanoparticles loaded with DOX through the endothelial cell and uptake by GB261 cells.

We then determined whether DOX taken up by GB261 cells was sufficient to induce apoptosis and ICD of these cells. Flow cytometry analysis of cells undergoing apoptosis (Figure 3(d)) showed that ENP_DOX_ treatment significantly increased the ratio of apoptotic cells compared to DOX or NP_DOX_ treatment (Figure 3(e)). Viability of cells was further determined by the MTT assay. Consistently, we found that ENP_DOX_ treatment significantly cell viability compared to DOX or NP_DOX_ treatment (Figure 3(f)). ICD was determined by surface exposure of CRT (Figure 3(g)), released HMGB1 in the supernatant of GL261 cell culture (Figure 3(h)) and ATP release (Figure 3(i)). Flow cytometry analysis showed that ENP_DOX_ treatment increased the number of cells with surface CRT among viable cells compared to DOX or NP_DOX_ treatment. Western blot analysis showed that the protein level of S‐HMGB1 was higher as a result of ENP_DOX_ treatment. Consistently, significantly more ATP was released from cells treated with ENP_DOX_ compared to other treatments. These results suggest that ENP_DOX_ induced a higher level of ICD among GB261 cells compared to DOX or NP_DOX_ treatment.

### 2.4 Systemic administration of END_DOX_ leads to efficient DOX accumulation in tumor tissue in a mouse model of GBM

**FIGURE 4:**
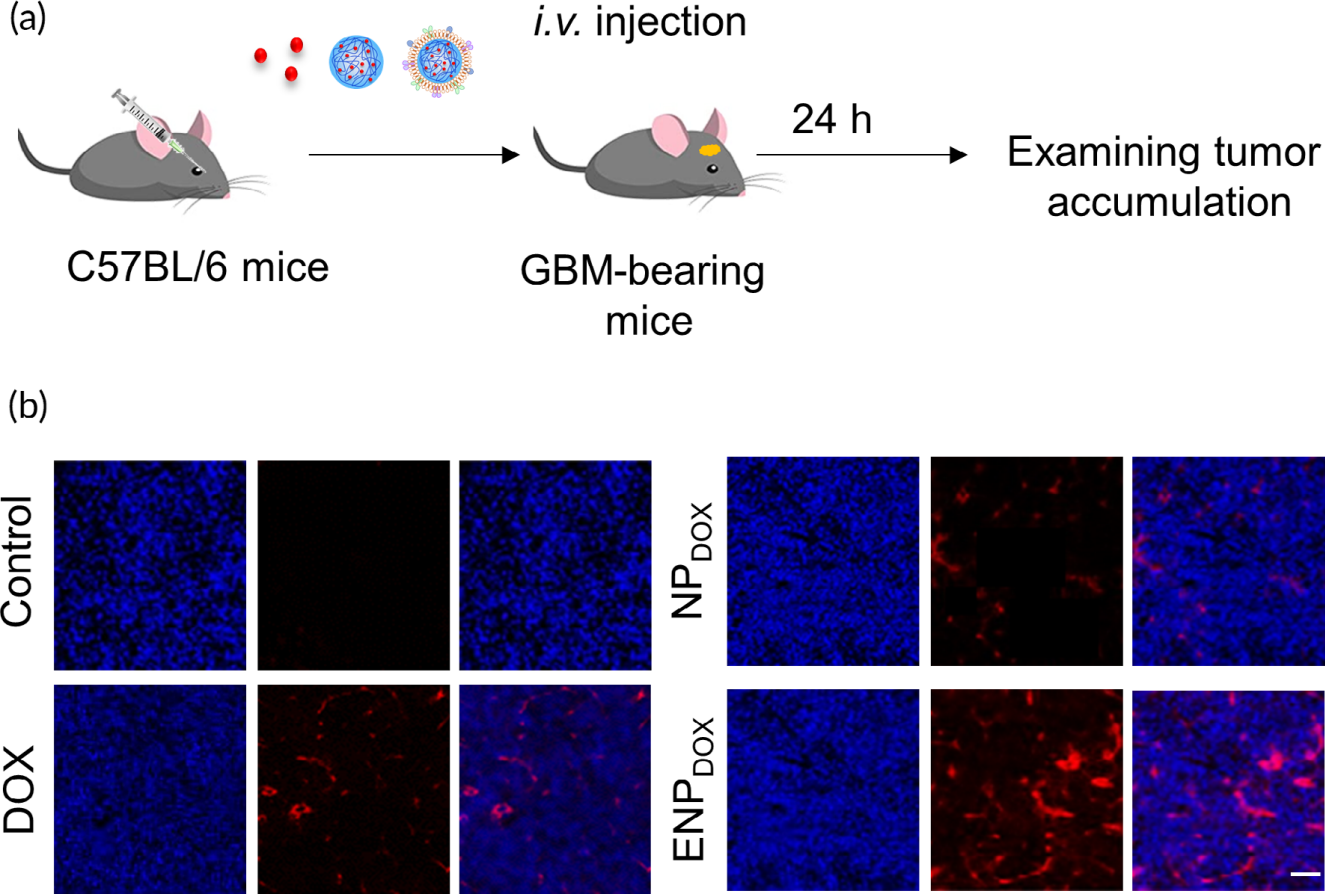
Glioblastoma accumulation of exosome‐coated doxorubicin (DOX)‐loaded nanoparticles (ENP_DOX_) in vivo. (a) Experimental protocol. Orthotopic glioblastoma xenograft model was established with GL261 cell line in 8–10‐week‐old C57BL/6 male mice. DOX, DOX‐loaded nanoparticles (NP_DOX_), or ENP_DOX_ were intravenous injected 14 days post‐tumor implantation. (b) Tumor accumulation of DOX‐loaded nanoparticles observed by confocal microscope. DAPI (blue) was used to stain the cell nucleus

To determine whether END_DOX_ administered systemically could penetrate the BBB and accumulate in the tumor tissue of the brain, we established an orthotopic GBM xenograft model by stereotactical implantation of GL261 cells into the brain as described previously.^17^ GBM‐bearing mice were injected with DOX, NP_DOX_, or END_DOX_ intravenously and DOX accumulation in the tumor tissues was examined 24 h later (Figure 4(a)). Although control tumor tissue showed no DOX fluorescent signal, tumor tissues isolated from mice treated with DOX and NP_DOX_ showed slight accumulation of DOX (Figure 4(b)). Importantly, tumor tissues from mice treated with ENP_DOX_ showed intense and widespread DOX signal, suggesting that exosome powerfully improved the efficiency of penetration of DOX‐loaded nanoparticles and delivery of the drug to tumor region.

### 2.5 ENP_DOX_ administration modulates the immune microenvironment of GBM‐bearing mice

**FIGURE 5:**
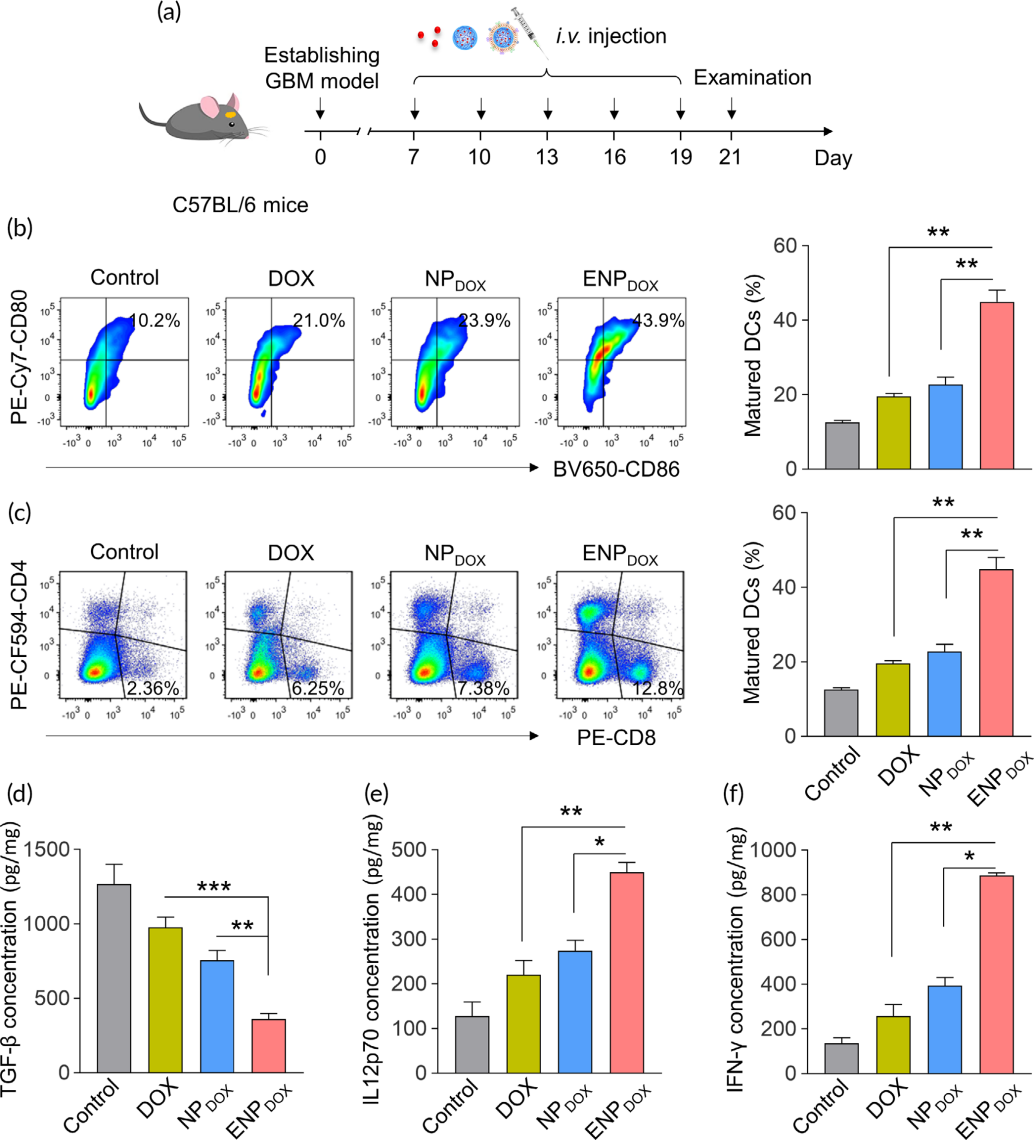
Exosome‐coated doxorubicin (DOX)‐loaded nanoparticles (ENP_DOX_) administration modulates the immune microenvironment of glioblastoma. (a) Orthotopic glioblastoma xenograft model was established with GL261 cell line in 8–10‐week‐old C57BL/6 male mice. DOX, DOX‐loaded nanoparticles (NP_DOX_), or ENP_DOX_ were intravenous injected 7 days post‐tumor implantation at indicated days. The tumor tissues were collected for analyzing the modulation of the immune microenvironment 2 days post last injection. (b) Flow cytometry gating and histogram analysis of matured dendritic cells (DCs) in deep cervical lymph nodes at the end of treatment. The matured DCs were denoted as CD80^+^CD86^+^ populations (gate in CD45^+^CD11b^+^CD11c^+^ cell population). (c) Flow cytometry gating and histogram analysis of cytotoxic T cells (CD8^+^ T cells) in the CD45^+^ tumor‐infiltrating lymphocytes (TIL) in tumor tissues from mice receiving indicated treatment. (d–f) ELISA results of cytokines production in the tumors from mice receiving indicated treatments (d: TGF‐β; e: IL12p70; f: IFN‐γ). Data represent means ± SD. *p < 0.05, **p < 0.01, ***p < 0.001

Orthotopic GBM xenograft model was established with GL261 cell line in 8–10‐week‐old C57BL/6 male mice (Figure 5(a)). To determine whether systemic administration of ENP_DOX_‐induced ICD in vivo, we examined maturation of dendritic cells in the deep cervical lymph nodes (Figure 5(b)) and the level of cytotoxic T cells in tumor tissues (Figure 5(c)). We found that compared to control, DOX and NP_DOX_ treatment, ENP_DOX_ significantly increased the ratio of mature dendritic cells (CD80^+^CD86^+^ cell population over CD45^+^CD11b^+^CD11c^+^ cell population) in GBM‐bearing mice following drug administration. Similarly, ENP_DOX_ also significantly increased the ratio of cytotoxic T cells among the CD45^+^ tumor‐infiltrating lymphocyte.

Examination of cytokine production in the tumors of drug treated mice also showed consistent results. ENP_DOX_ significant reduced TGF‐ß (Figure 5(d)) production and increased IL12p70 (Figure 5(e)) and IFN‐γ (Figure 5(f)) production.

These results suggest that systemic administration of ENP_DOX_ is sufficient to induce changes in the immune microenvironment of GBM‐bearing mice.

### 2.6 ENP_DOX_ administration prolongs the survival of GBM‐bearing mice

**FIGURE 6:**
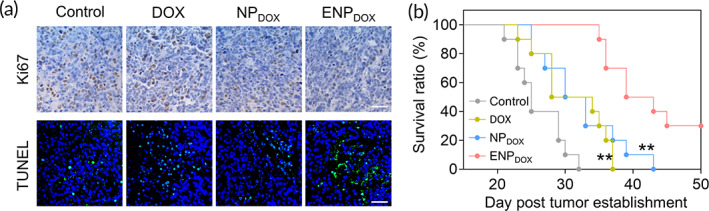
Exosome‐coated doxorubicin (DOX)‐loaded nanoparticles (ENP_DOX_) administration prolongs the survival of glioblastoma‐bearing mice. (a) Representative images of immunobiological staining for Ki67 and immunofluorescence staining of TUNEL in tumor section from different treated mice. Tumor tissues were collected 2 days post last injection. (b) Kaplan–Meier survival curves of glioblastoma‐bearing mice receiving different treatments. Data represent means ± SD. **p < 0.01 (ENP_DOX_ vs. DOX and DOX‐loaded nanoparticles [NP_DOX_])

Finally, we investigated whether systemic administration of ENP_DOX_ was able to suppress tumor growth and improve the survival of GBM‐bearing mice. Staining of tumor sections with Ki67, a marker of cell proliferation, showed that mice treated with ENP_DOX_ had reduced proliferating cells (Figure 6(a), top panels). TUNEL staining of apoptotic cells showed that tumor tissues had increased apoptotic cells following ENP_DOX_ treatment compared to control, DOX, or NP_DOX_ treatment (Figure 6(a), bottom panels).

To determine the effect of ENP_DOX_ on survival, GBM‐bearing mice were treated with DOX, NP_DOX_, or ENP_DOX_ and their survival duration was recorded (Figure 6(b)). Kaplan–Meier survival analysis showed that both DOX and NP_DOX_ treatments slightly extended the survival of GBM‐bearing mice compared to control mice. Importantly, mice treated with ENP_DOX_ survived significantly longer compared to that treated with DOX and NP_DOX_.

## 3 DISCUSSION

GBM is a highly aggressive brain cancer with extremely poor prognosis. Unfortunately, treatment of GBM is largely impeded by the invasiveness of the treatments or the inefficiency of drugs in penetrating the BBB to reach the tumor locations. Recent advancement in drug delivery system has provided a potential avenue for delivery of drugs across the BBB in the treatment of neurological diseases. In this study, we investigated the effect of exosome coating in promoting penetration of DOX‐loaded nanoparticles and the potential of DOX in inducing apoptosis and ICD of glioma cells for effective treatment of GBM. We prepared ENP_DOX_ that express proteins relevant to exosomes including CD9, CD63, CD81, and HSP70. Mouse brain endothelial bEnd.3 cells could effectively take up DOX when treated with ENP_DOX_. In an in vitro BBB assay, we showed that ENP_DOX_ was able to penetrate mouse endothelial cells and induce apoptosis and ICD of glioma cells, including increased surface CRT exposure, HMGB1 secretion and ATP release, manifestations that are characteristics of ICD. In an in vivo mouse model of GBM, our results showed that intravenous administration of ENP_DOX_ lead to DOX accumulation in the GBM xenograft. DOX delivered through ENP_DOX_ significantly induced maturation of dendritic cells in cervical lymph nodes and activation of cytotoxic T lymphocytes as well as production of proinflammatory cytokines. Importantly, our study showed that systemic ENP_DOX_ suppressed proliferation and increased apoptosis of tumor cells and significantly prolonged survival of GBM‐bearing mice.

The BBB has been a great obstacle in the treatment of various neurological disorders, including GBM, with only small molecules being able to penetrate through the BBB. Most available drugs are unable to transition from the blood circulation to the brain, making systemic administration impossible for treating brain diseases.^18^ The objective of drug development in the GBM treatment is to search for drugs with assistant from drug delivery systems to penetrate the BBB and target glioma cells. Several strategies have been exploited for delivering drugs to brain including viral vectors, nanoparticles, and brain permeability enhancers, with each method having its advantages and limitation, such as safety concerns and toxicity. Recently, exosomes have been actively studies as a promising vector for drug delivery in various cancer types.^19^ For example, exosomes derived from macrophages have been specifically engineered to deliver drugs that can target tumors, leading to inhibition of tumor growth and increases apoptosis of tumor cells.^13^ In a zebrafish brain cancer models, exosomes are used for delivery of anticancer drug which suppressed markers of tumor growth.^20^

In our study, we purified exosomes derived from murine brain endothelial cells and used them for coating nanoparticles loaded with the anticancer drug DOX. The removing of contents inside the vesicles and adding DOX‐loaded nanoparticles did not change the expression of exosome membrane proteins. In addition to being taken up in brain endothelial cells, our study showed that ENP_DOX_ was effectively taken up by glioma cells both in an in vitro model of BBB and an in vivo model of GBM. Our findings suggest that exosomes may be an effective vehicle for delivering drugs across the BBB and targeting brain tumors. Importantly, in the mouse model of GBM, we administered ENP_DOX_ through intravenous injection and this systemic delivery of drug loading nanoparticles allowed effective targeting of the anticancer drug to GBM. Consistent with our study, exosomes have been used as a carrier of siRNA which promoted efficient systemic delivery of siRNA to brain regions.^21^ The previous studies together with ours suggest that exosome can be used to target drugs to the brain through noninvasive systemic administration.

Previously, engineered exosomes derived from macrophages have been used for coating nanoparticles loading DOX and have been shown to induce potent apoptosis of breast cancer cells and inhibition of tumor growth.^13^ In addition to inducing apoptosis, DOX is also an inducer of ICD and this property has been utilized in the studies of cancer therapy for antitumor immunity. DOX treatment leads to immuomodulation following death of tumor cells in a mouse model of neuroblastoma.^22^ After confirming efficient uptake of DOX delivered through ENP_DOX_ by glioma cells, our study focused on investigating the efficacy of this anticancer drug in tumor suppression in order to determine its potential in the treatment of GBM. We showed that ENP_DOX_ induced apoptosis and ICD of glioma cells in vitro with increased CRT surface exposure and increased release of HMGB1 and ATP. Consistently, END_DOX_‐induced dendritic cell maturation and cytotoxic T lymphocyte activation in GBM‐bearing mice. Our study not only confirmed the previous findings of DOX in tumor suppression by inducing apoptosis and ICD, but also successfully achieved penetration of DOX through the BBB using the exosome‐mediated drug delivery system. The finding that systemic administration of ENP_DOX_ significantly prolonged the survival of GBM‐bearing mice is of great clinical importance for the treatment of GBM since GBM is the deadliest brain cancer and patients with GBM have an extremely short average survival with a 5‐year survival rate of approximately 5%.^23^

Nanomedicines in cancer field have been designed to target tumors with an intention to reduce side effects.^24^ DOX‐loading nanoparticles (Doxil) has been approved by FDA for treating various cancers including ovarian cancer, HIV‐associated Kaposi's sarcoma, and other cancers.^24^ Some side effects were reported such as skin toxicity with less frequent cases with mild cardiotoxicity and hepatotoxicity.^25^ In this study, we have not investigated the impact of on ENP_DOX_ healthy cells. Survival analysis of mice bearing GBM treated with ENP_DOX_ showed that ENP_DOX_ significantly prolonged the survival of the mice, suggesting that ENP_DOX_ effectively suppressed GBM growth without causing any devastating side effects. However, detailed analysis of the side effects will be carried out in the future studies for clinical application.

In summary, our study revealed a potent drug delivery system by coating DOX‐loaded nanoparticles with brain endothelial cell derived exosomes to facilitate penetration of anticancer drugs across the BBB and target the GBM. We have shown that ENP_DOX_ induced apoptosis and ICD both in vitro and in vivo, and it significantly prolonged the survival of GBM‐bearing mice. Our important findings warrant further investigation of this system in clinical application.

## 4 MATERIALS AND METHODS

### 4.1 Preparation and characterization of exosome‐coated DOX‐loaded nanoparticles

DOX‐loaded nanoparticles were prepared using an emulsion method as described previously.^15^ Briefly, PEG–PLA and DOX solution in dimethyl sulfoxide (DMSO) and chloroform was emulsified in water for an oil‐in‐water emulsion. Chloroform was then removed by evaporation and DMSO was removed by dialyzing the resulting production against water. Free DOX was removed by centrifugation. Nanoparticles encapsulating DOX were denoted as NP_DOX_.

Exosomes derived from endothelial cells were isolated with the contents removed according to a previous study.^13^ NP_DOX_ were packaged into the empty exosomes by mixing the NP_DOX_ with the exosomes. The exosome‐coated NP_DOX_ (denoted as ENP_DOX_) were obtained by extruding the mixture through a 100 nm polycarbonate porous membrane.

The morphology and diameter of NP_DOX_ and ENP_DOX_ were determined by transmission electron microscopy. Zeta potential was measured through dynamic light scattering.^13^

### 4.2 Mice

C57BL/6 mice were acquired from Cyagen Biosciences Inc (Suzhou, China) and housed in an environmentally controlled animal facility with free access to food and water. Orthotopic GBM xenograft model was established in 8–10‐week‐old C57BL/6 male mice by stereotactical implantation of 50,000–250,000 GL261 cells as described previously.^17^ To determine DOX accumulation in the tumor tissue, mice were treated with free DOX, NP_DOX_, or ENP_DOX_ through intravenous injection at 14 days following tumor implantation. Accumulation of DOX in the tumor tissue was examined at 24 h after drug treatment. To determine the immune microenvironment of GBM, mice were treated with free DOX, NP_DOX_, or ENP_DOX_ at days 7, 10, 13, 16, and 19 following tumor implantation and tumor tissues were harvested for subsequent analysis at Day 21. Tumor cell proliferation and apoptosis were determined by Ki67 and TUNEL staining of tumor tissue isolated 2 days after the last drug injection. All procedures were approved by the ethics committee of the Second Hospital of Hebei Medical University.

### 4.3 Western blot

Expression of exosome relevant proteins including CD9, CD63, CD81, and HSP70, and level of released HMGB1 in the supernatant of GL2261 cell culture was assessed by Western blot analysis. Briefly, protein preparations were separated by SDS‐PAGE electrophoresis, transferred onto membranes, blocked with 2% milk for 2 h, incubated in primary antibodies at 4°C overnight and secondary antibodies for 1 h at room temperature after washing. Proteins were developed with chemiluminescent reagents.

### 4.4 In vitro BBB model

The transwell system was adopted to study penetration of ENP_DOX_ through the BBB in vitro as described previously.^16^ Briefly, the murine brain endothelial bEnd.3 cells were cultured in the upper chamber to mimic the BBB in vitro and the GL261 cells, a line of glioma cells, were cultured in the lower chamber. Free DOX, NP_DOX_, and ENP_DOX_ were added to the upper compartment respectively for 12 h and the cellular uptake of DOX in GL261 cells was assessed subsequently. GL261 cells were cultured for additional 24 h before examination of apoptosis and ICD.

### 4.5 Flow cytometry

To determine the uptake of DOX by mouse brain endothelial bEnd.3 cells, bEnd.3 cells were incubated with free DOX, NP_DOX_, or ENP_DOX_ with a concentration of DOX at 0.5 μg/mL for 4 h and the fluorescent intensity of DOX was assessed through a flow cytometer. Similarly, cellular uptake of DOX in GL261 glioma cells following transwell culture was also assessed by flow cytometry.

To determine apoptosis of GL261 cells following transwell culture, GL261 cells were stained with Annexin‐V and propidium iodide for 15 min according to manufacturer's instruction and were subjected to flow cytometry.

To determine surface exposure of CRT, GL261 cells following transwell culture were stained with propidium iodide and FITC‐labeled anti‐CRT antibody and CRT positive cells were counted through flow cytometry.

Mature dendritic cells were determined by the ratio of CD80^+^CD86^+^ cells gated in CD45^+^CD11b^+^CD11c^+^ population in deep cervical lymph nodes and cytotoxic T cells were determined by ratio of CD8^+^ cells among CD45^+^ tumor‐infiltrating lymphocytes in tumor tissue as described previously.^26^ Deep cervical lymph nodes and tumor tissues were isolated at 2 days following intravenous injection of free DOX, NP_DOX_, or ENP_DOX_ and digested with Type 4 collagenase for about 1.5 h to form a single cell suspension. Cells were then stained with relevant antibodies and were subjected to flow cytometry analysis.

All antibodies used for flow cytometric analysis were bought from Biolegend, and a 1:400 dilution was used for examination.

### 4.6 In vitro and in vivo uptake of DOX

bEnd.3 cells were treated with DOX, NP_DOX_, or ENP_DOX_ for 1 h. Nuclei were stained with DAPI for 10 min. Intracellular uptake of DOX in bEnd.3 cells was then assessed by confocal laser scanning microscopy (CLSM).

To determine uptake of DOX in the tumor tissue, orthotopic GBM xenograft mice were established and were treated with intravenous injection of DOX, NP_DOX_, or ENP_DOX_. One day after indicated treatment, tumors were isolated, sectioned, stained with DAPI, and DOX accumulation in the tumor tissue were assessed by CLSM.

### 4.7 ELISA

ATP release from GL261wells following transwell culture was determined by ELISA using a chemiluminescent ATP Determination Kit according to manufacturer's instruction (Invitrogen™, cat# A22066). Cytokines produced from the tumors of mice treated with intravenous DOX, NP_DOX_, or ENP_DOX_ were examined using ELISA assay kits following manufacturer's instruction.

### 4.8 MTT assay

Cell viability was determined by the MTT assay using an MTT assay kit (Sigma) according to manufacturer's instruction.

### 4.9 Statistical analysis

Eight mice were used per group for animal study. All in vitro experiments were repeated three times independently. Data were analyzed by SPSS. Differences between two groups were determined by Student's *t*‐test or one‐way ANOVA analysis followed by a Tukey's post hoc test. Kaplan–Meier survival curves were used to determine the differences in the survival of GBM‐bearing mice receiving different treatments. Differences with *p* < 0.05 were considered statistically significant.

## AUTHOR CONTRIBUTIONS

**Chao Zhang:** Data curation; formal analysis. **Jian Song:** Data curation; software. **Lei Lou:** Data curation; validation. **Xuejiao Qi:** Data curation; validation. **Lei Zhao:** Data curation; validation. **Bo Fan:** Data curation; resources; software. **Guozhu Sun:** Data curation; resources; software. **Zhongqiang Lv:** Validation; visualization. **Zhenzeng Fan:** Supervision; validation. **Baohua Jiao:** Formal analysis; methodology; resources; supervision. **Jiankai Yang:** Conceptualization; investigation; resources; supervision; writing‐original draft; writing‐review and editing.

## CONFLICT OF INTEREST

No conflicts of interest, financial or otherwise, are declared by the authors.

## Data Availability

Data for generating the figures could be obtained with reasonable request to Dr. Jiankai Yang.
