# Correction to “Doxorubicin‐loaded nanoparticle coated with endothelial cells‐derived exosomes for immunogenic chemotherapy of glioblastoma”

**DOI:** 10.1002/btm2.10719

**Published:** 2024-09-05

Zhang C, Song J, Lou L, et al. Doxorubicin‐loaded nanoparticle coated with endothelial cells‐derived exosomes for immunogenic chemotherapy of glioblastoma. Bioeng Transl Med 2020;6(3):e10203.

The three images of NP_DOX_ group in Figure 4b were incorrect as follows.

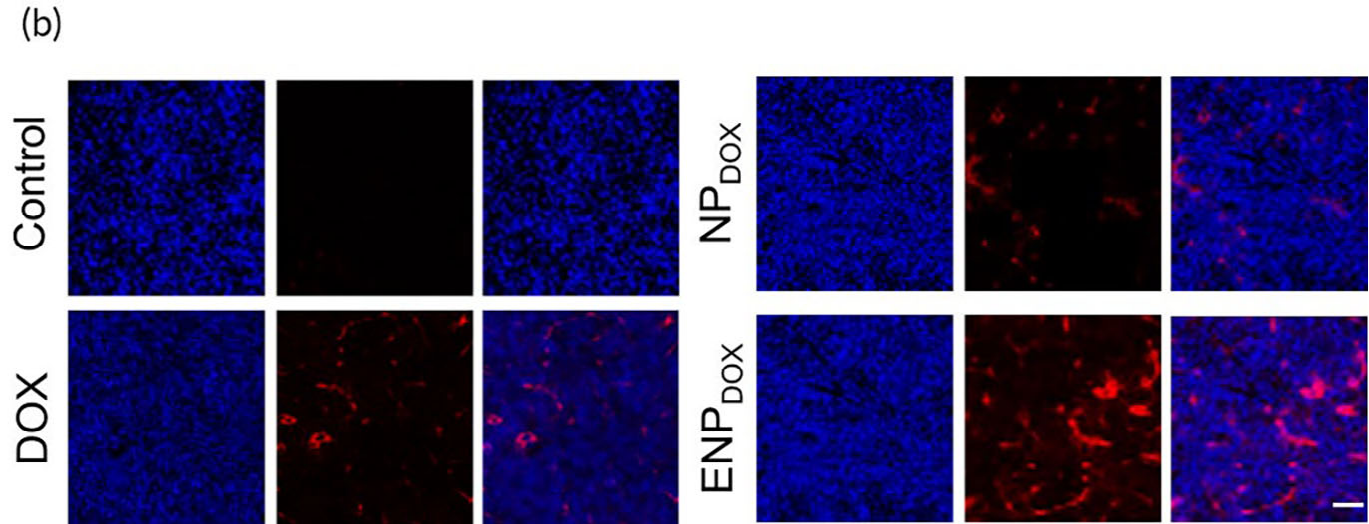

They should have displayed as below.

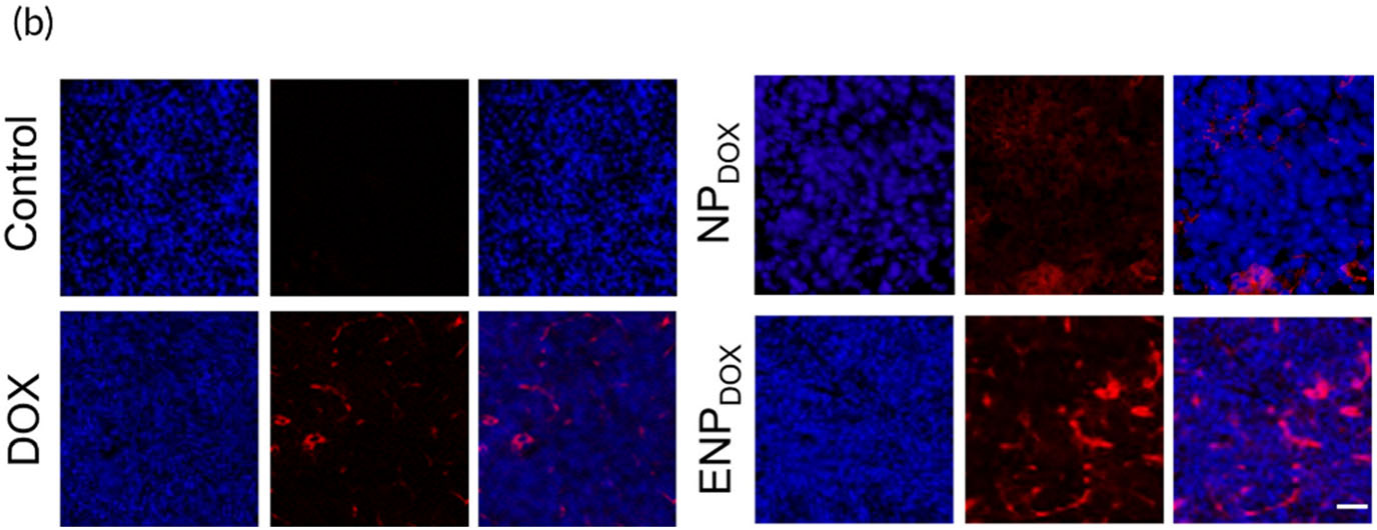

We apologize for this error.

